# Supplementary material for: Simulations and active learning enable efficient identification of an experimentally-validated broad coronavirus inhibitor
Source: Nat Commun. 2025 Jul 29;16:6949. doi: 10.1038/s41467-025-62139-5 (PMC12307812; doi:10.1038/s41467-025-62139-5)
Supplement: Supplementary file 1 — Supplementary Information [file 41467_2025_62139_MOESM1_ESM.pdf]

Supplementary information: SIMULATIONS AND ACTIVE LEARNING ENABLE EFFICIENT IDENTIFICATION OF AN EXPERIMENTALLY-VALIDATED BROAD CORONAVIRUS INHIBITOR

Katarina Elez[1], Tim Hempel[1,2,3], Jonathan H. Shrimp[4], Nicole Moor[5,6], Lluís Raich[1], Cheila Rocha[5,6], Robin Winter[1,7], Tuan Le[1,7], Stefan Pöhlmann[5,6], Markus Hoffmann[5,6], Matthew D. Hall[4], and Frank Noé[1,2,3,8,*]

[1]Department of Mathematics and Computer Science, Freie Universität Berlin, Berlin, Germany
[2]Department of Physics, Freie Universität Berlin, Berlin, Germany
[3]Microsoft Research AI for Science, Berlin, Germany
[4]National Center for Advancing Translational Sciences, National Institutes of Health, Rockville, MD, USA
[5]Infection Biology Unit, German Primate Center - Leibniz Institute for Primate Research, Göttingen, Germany
[6]Faculty of Biology and Psychology, University Göttingen, Göttingen, Germany
[7]Department of Bioinformatics, Bayer AG, Berlin, Germany
[8]Department of Chemistry, Rice University, Houston, TX, USA

* Corresponding author: franknoe@microsoft.com

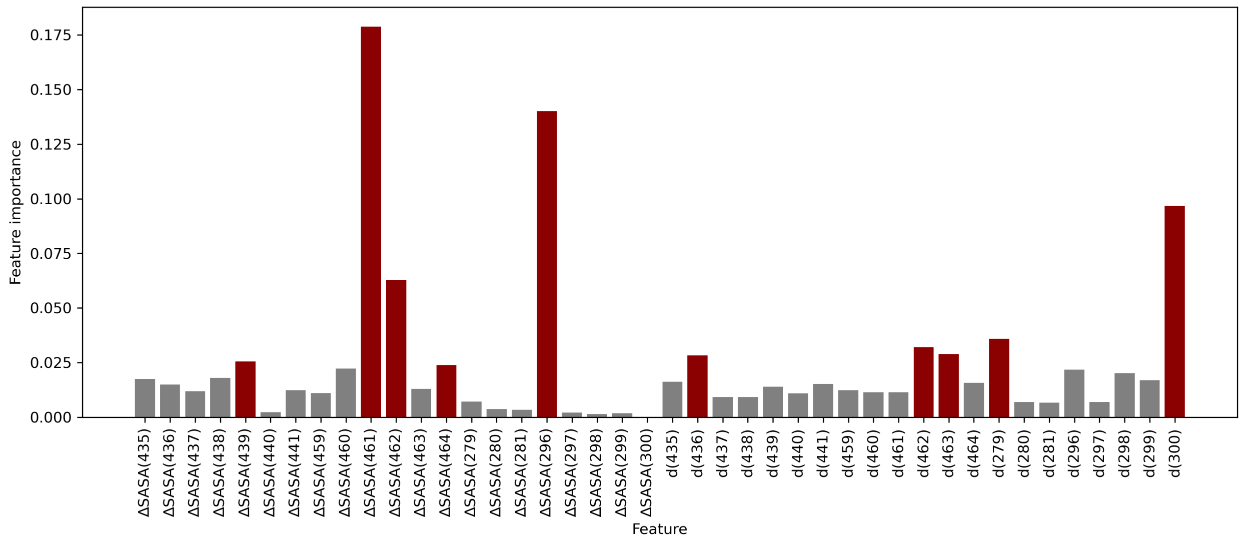

Figure S1: Feature importance from the random forest regressor trained to predict the trypsin-domain-specific score. Residues are numbered according to TMPRSS2. Top 10 most important features are highlighted in red.

Rank	ID	Dynamic h-score
#1	DB03417	1.443
#2	DB00202	1.311
#3	**DB12598**	1.308
#4	**DB13729**	0.963
#5	DB07366	0.937
#6	DB12645	0.936
#7	DB03536	0.881
#8	DB07985	0.849
#9	DB06861	0.798
#10	DB00449	0.781
#11	**DB12831**	0.721
#12	DB04424	0.700
#13	DB08732	0.683
#14	DB01161	0.683
#15	**DB06635**	0.681

Table S1: Top 15 DrugBank compounds ranked by their h-score. Entries for nafamostat, camostat, gabexate and otamixaban are bolded, respectively.

DB03417

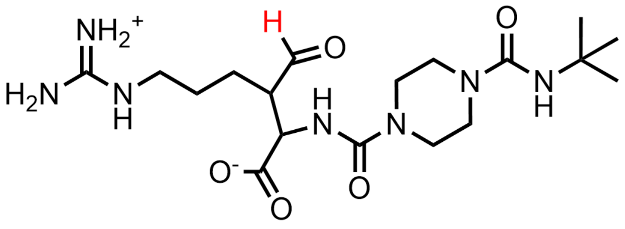

BMS-262084

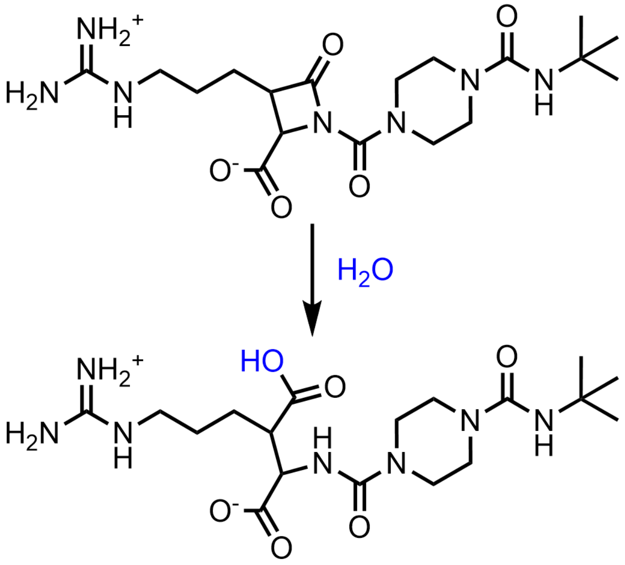

Figure S2: Chemical relation between DB03417 and BMS-262084.

1 - NCGC00378763-01 - 0.79 µM

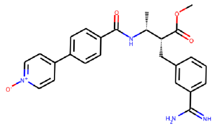

2 - NCGC00378882-01 - 2.24 µM

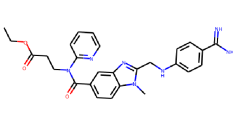

3 - NCGC00417192-01 - 8.91 µM

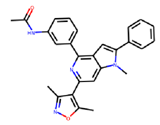

4 - NCGC00411596-01 - 8.91 µM

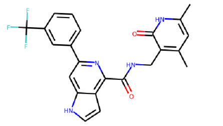

5 - NCGC00102811-01 - 12.59 µM

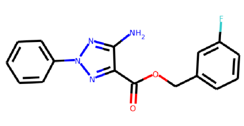

6 - NCGC00100604-01 - 12.59 µM

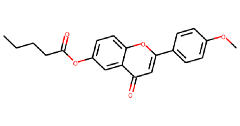

7 - NCGC00424555-01 - 12.59 µM

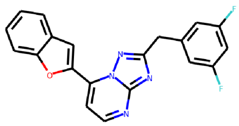

8 - NCGC00138820-01 - 12.59 µM

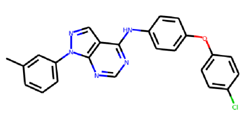

9 - NCGC00127764-01 - 14.13 µM

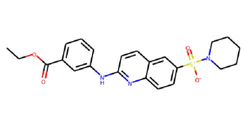

10 - NCGC00103995-01 - 14.13 µM

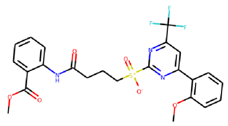

11 - NCGC00116348-01 - 15.85 µM

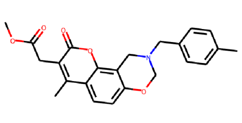

12 - NCGC00476320-01 - 15.85 µM

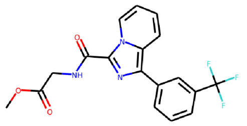

13 - NCGC00099485-01 - 15.85 µM

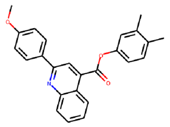

14 - NCGC00099825-01 - 17.78 µM

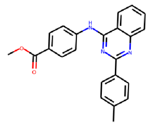

15 - NCGC00411690-01 - 17.78 µM

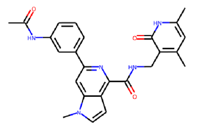

16 - NCGC00263161-11 - 19.95 µM

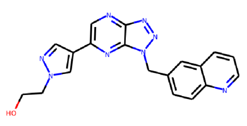

17 - NCGC00434595-01 - 19.95 µM

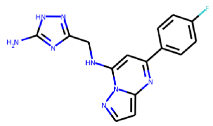

18 - NCGC00099237-01 - 22.39 µM

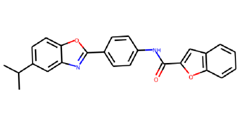

19 - NCGC00117127-01 - 22.39 µM

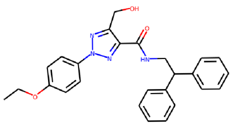

20 - NCGC00249912-01 - 31.62 µM

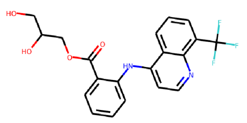

21 - NCGC00485933-01 - 31.62 µM

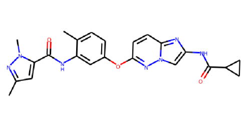

22 - NCGC00099773-01 - 31.62 µM

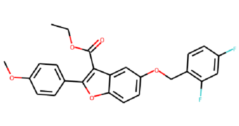

23 - NCGC00104930-01 - 31.62 µM

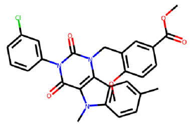

24 - NCGC00411713-01 - 35.48 µM

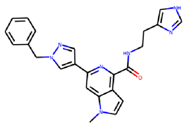

25 - NCGC00482851-02 - 35.48 µM

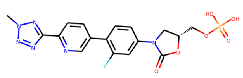

26 - NCGC00413991-01 - 35.48 µM

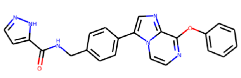

Figure S3: Compounds with maximum response below -40% from the first round of screening on the NCATS in-house library sorted by ascending IC50 value.

1 - NCGC00253593-01 - 14.13 μM

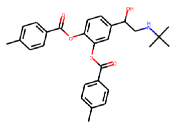

2 - NCGC00127812-01 - 15.85 μM

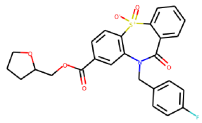

3 - NCGC00102940-01 - 17.78 μM

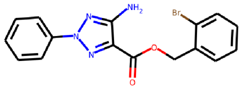

4 - NCGC00102837-01 - 17.78 μM

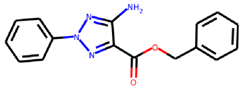

5 - NCGC00102929-01 - 17.78 μM

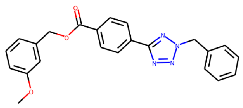

6 - NCGC00417822-01 - 19.95 μM

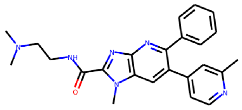

7 - NCGC00415676-01 - 31.62 μM

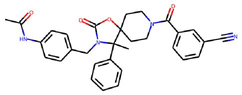

Figure S4: Compounds with maximum response below -40% from the second round of screening on the NCATS in-house library sorted by ascending IC50 value.

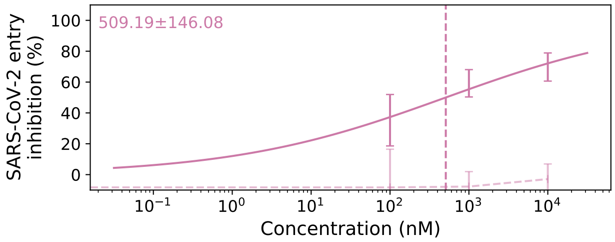

Figure S5: Dose-response curve and IC50 estimate for inhibition of live SARS-CoV-2 infection of Calu-3 cells. The average (mean) ± SD of three biological replicates is shown.

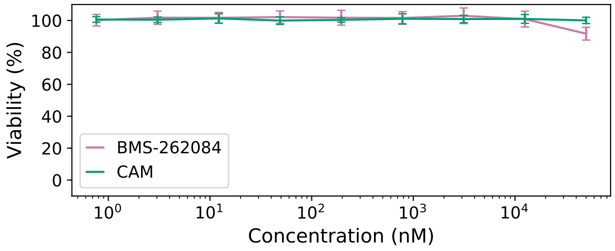

Figure S6: Effect of inhibitors on Calu-3 cell viability. The average (mean) ± SD of two biological replicates is shown. Each biological replicate was performed with four technical replicates.

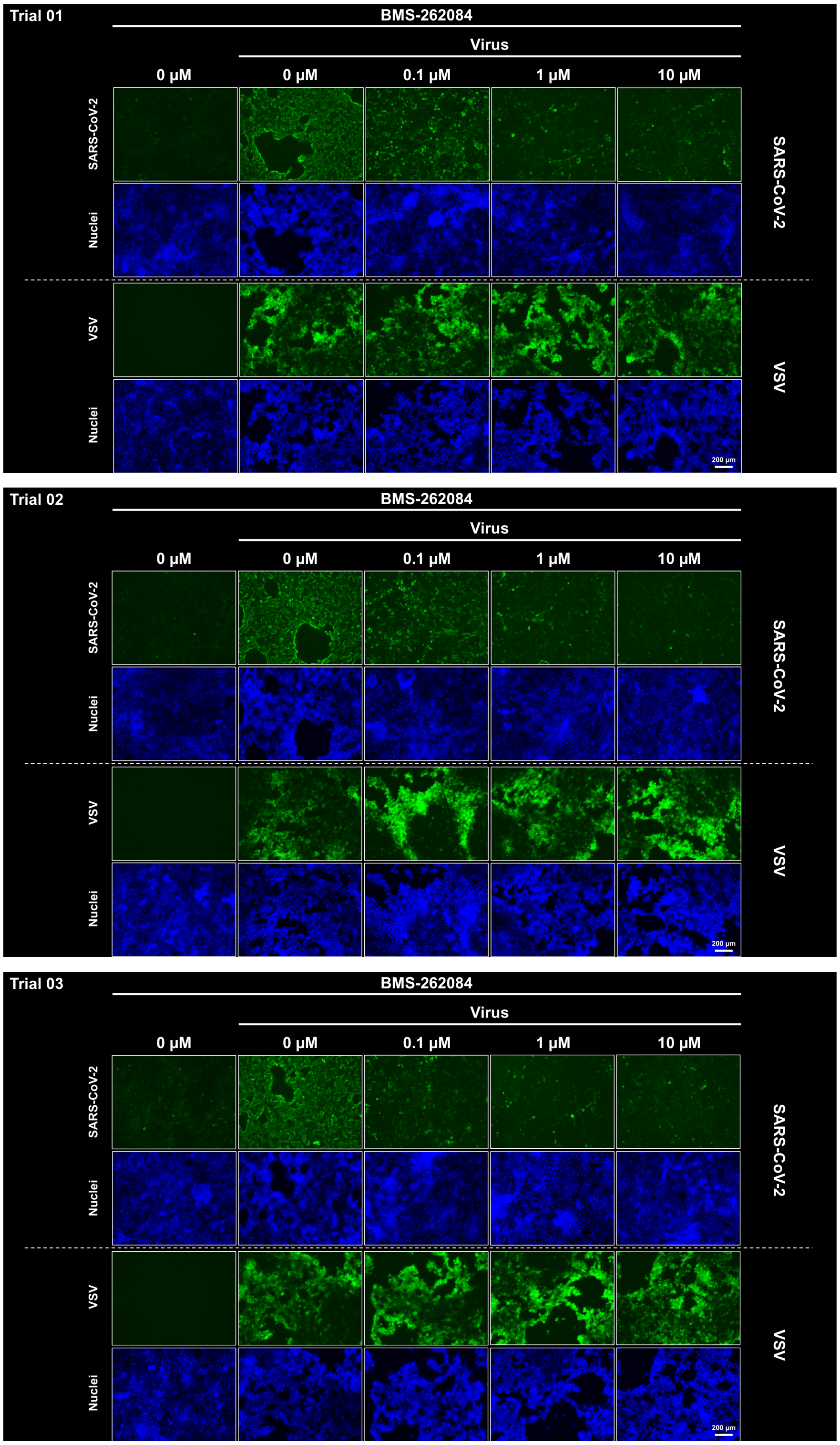

Figure S7: Microscopic images of BMS-262084-treated Calu-3 cells infected by live SARS-CoV-2 (upper) or VSV (lower). Green indicates SARS-CoV-2/VSV-positive (infected) cells and blue indicates nuclei. Three biological replicates are shown.

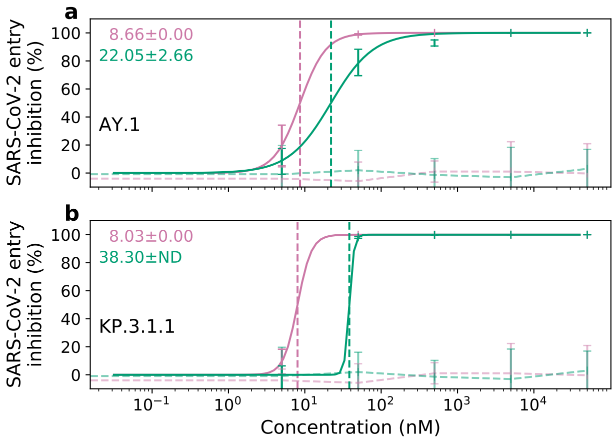

Figure S8: Dose-response curves and IC50 estimates for inhibition of live SARS-CoV-2 infection of Calu-3 cells. The average (mean) ± SD of three biological replicates is shown. Note that the IC50 uncertainty for camostat inhibiting KP.3.1.1 could not be reliably estimated due to the steepness of the curve (ND = not determined).

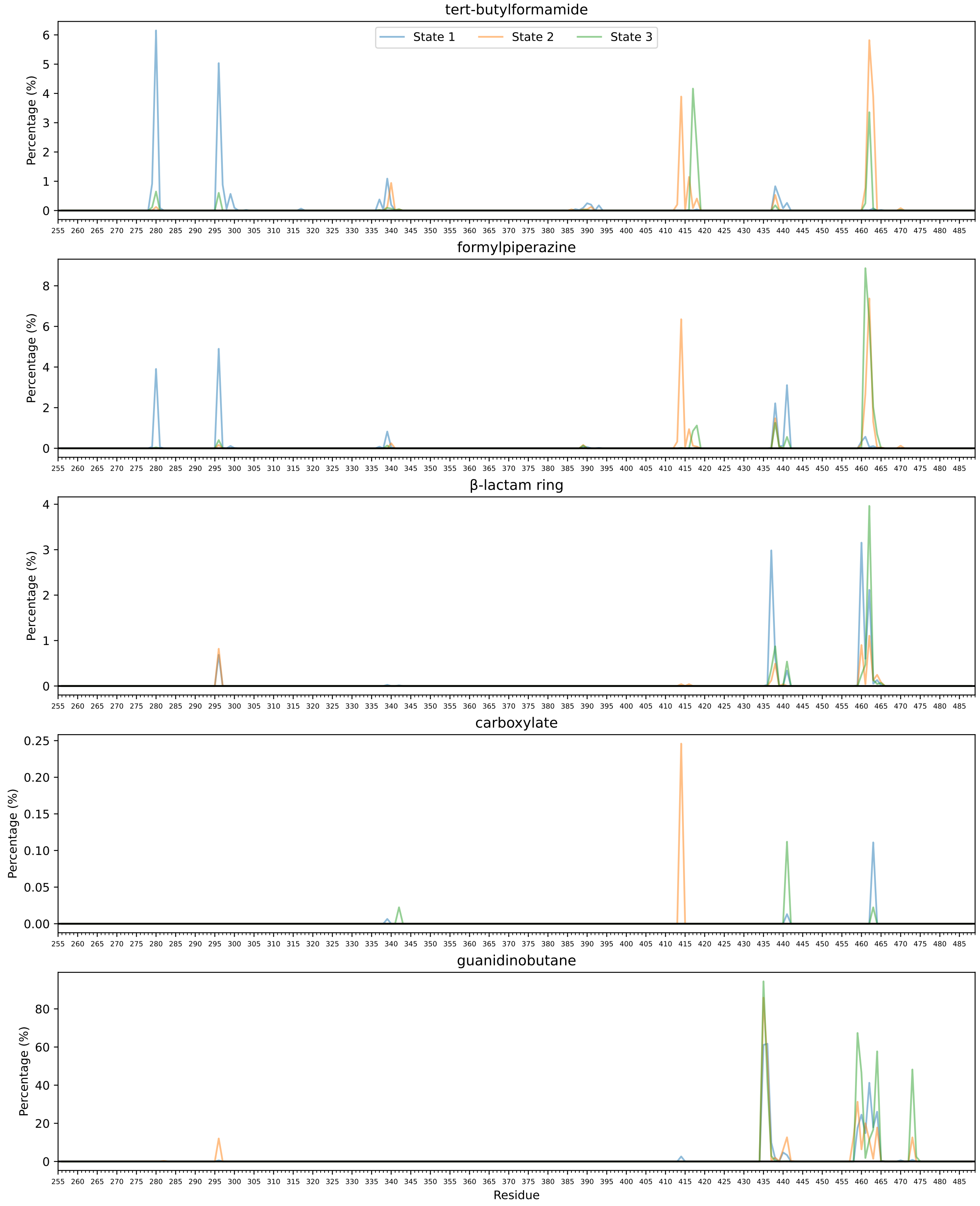

Figure S9: Contact statistics for the three metastable binding modes. For each drug group, percentage of frames in which that group is within 2.5 Å from each residue is shown.

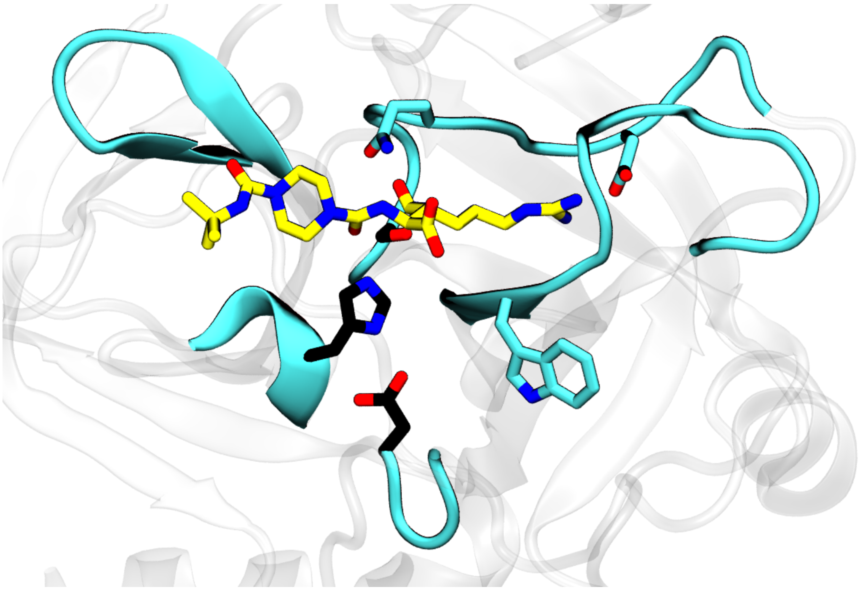

Figure S10: BMS-262084 in a covalent complex with bovine trypsin (PDB ID: 1RXP).

Algorithm S1 Active learning cycle.

```
procedure INIT(library, n_clusters)
    fingerprints ← getMorganFingerprints(library)
    clusters ← kmeans(fingerprints, n_clusters)
    ini ← getRepresentatives(clusters)
    screened ← ini
    scores ← score(ini)
    return screened, scores
end procedure

procedure EXTEND(encs, screened, scores, potential, ext_size)
    encs_screened ← encs[screened]
    model ← SVR()
    train(model, encs_screened, scores)
    encs_potential ← encs[potential]
    pred ← predict(model, encs_potential)
    ext ← getBest(pred, ext_size)
    scores_ext ← score(ext)
    screened ← screened ∪ ext
    scores ← scores ∪ scores_ext
    return screened, scores
end procedure

procedure MAIN(library, n_clusters, ext_size)
    screened, scores ← init(library, n_clusters)
    encs ← getCDDDEncodings(library)
    i ← 1
    while i ≤ n_rounds do
        potential ← library \ screened
        screened, scores ← extend(encs, screened, scores, potential, ext_size)
        i ← i + 1
    end while
    return screened, scores
end procedure
```
